# More massive but potentially less healthy: black vultures feeding in rubbish dumps differed in clinical and biochemical parameters with wild feeding birds

**DOI:** 10.7717/peerj.4645

**Published:** 2018-04-19

**Authors:** Pablo Ignacio Plaza, Sergio Agustin Lambertucci

**Affiliations:** Grupo de investigaciones en Biología de la Conservación, Laboratorio Ecotono, INIBIOMA (Universidad Nacional Del Comahue—CONICET), San Carlos de Bariloche, Argentina

**Keywords:** Biochemical parameters, Landfill, Black vultures, Health studies, Nutritional problems

## Abstract

**Background:**

Organic waste is one of the most important anthropogenic food subsidies used by different species. However, there is little information about the health impact that rubbish dumps produce on species foraging in these sites.

**Methods:**

We studied the effect that rubbish dumps produce on the health of a scavenging bird from the Americas, the black vulture (*Coragyps atratus*). We sampled and studied clinical and biochemical parameters in 94 adult black vultures from two different sites in North Western Patagonia, a rubbish dump and the wild steppe.

**Results:**

We found differences in clinical and biochemical parameters between sites. Body mass was greater in individuals from the dump, whereas in the steppe there were more individuals clinically dehydrated. Biochemical parameters such as uric acid, calcium, alkaline phosphatase, glycaemia, globulins and haematocrit had higher values in individuals using the dump than in individuals from the steppe. Other biochemical parameters such as aspartate aminotransferase, alanine aminotransferase, creatine phosphokinase and urea were higher in individuals from the steppe than in individuals from the dump.

**Discussion:**

Foraging in organic waste could be considered beneficial for black vultures because they increase body mass and parameters associated to nutritional status like calcium and haematocrit. However, foraging in dumps can also affect their health status due to nutritional problems, potential kidney damage or infections that are signalled by the higher values of glycaemia, uric acid and globulins found in individuals from the dump. Our results highlight the contrasting effects that rubbish dumps may produce on wildlife health. They are relevant to different species using these sites, and are also an additional instrument for managing waste.

## Introduction

Using rubbish dumps as a food resource can produce both, positive and negative impacts on wildlife. This food source may improve body condition, reproductive fitness, population survival and even increase population abundance (Oro et al., 2013; Plaza & Lambertucci, 2017). However, foraging in rubbish dumps may have a negative impact on individuals, such as increasing the risk of infections with pathogens and poisoning with a great variety of toxics (Plaza & Lambertucci, 2017). Some studies call attention to the nutritional quality of this kind of food, suggesting that it could be considered a bad quality source producing impacts which are difficult to predict (Annett & Pierotti, 1989; Steigerwald et al., 2015). Therefore, a foraging strategy focused on rubbish dumps appears to be positive in some aspects but may be harmful in others.

Nowadays different toxics, pathogens and poor quality food sources (e.g. with bacteria, drug residues or foreign bodies) can be considered important causes of health impact, mortality and biodiversity loss (Daszak, Cunningham & Hyatt, 2000; Deem, Karesh & Weisman, 2001; Acevedo-Whitehouse & Duffus, 2009; Rideout et al., 2012). Therefore, the determination of medical variables in species that exploit rubbish dumps and could be exposed to these threats may provide important information to infer the potential effects that these sites may produce on wildlife (Bird et al., 2007; Hernández & Margalida, 2010; Plaza & Lambertucci, 2017). In this sense, health monitoring of species using these sites could be considered an early-warning system that can help to diagnose different pathologic processes or threats that could be affecting them (Bird et al., 2007; Ryser-Degiorgis, 2013). Moreover, the information produced by health studies in species using organic waste as a food source could be very useful to implement waste management and conservation policies for these areas and the different species that exploit these sites (Ryser-Degiorgis, 2013).

Scavenger birds have been sympatric and have interacted with humans for over millions of years (Morelli et al., 2015). They are particularly associated with habitats like rubbish dumps due to the high predictability and availability of food that they exploit (Pomeroy, 1975; Gangoso et al., 2012). In fact, evidence reveals that an increase in scavenger population could be associated to the use of anthropogenic habitats (Gangoso et al., 2012; Plaza & Lambertucci, 2017). The black vulture (*Coragyps atratus*) is an avian scavenger that exploits rubbish dumps throughout the Americas (Iñigo Elias, 1987; Sazima, 2013). However, there is no information about the health effects that this foraging strategy can produce in this species. This is relevant given that black vultures could be an indicator species for endangered scavengers using these sites, due to their large populations and because they are easier to sample (Caro & O’doherty, 1999).

The aim of this study is to determine if foraging in rubbish dumps affects the health of black vultures in the North-Western Argentine Patagonia. We hypothesize that the characteristics of the diet present at these sites produce changes in clinical and biochemical parameters that reflect health impacts. Therefore, we predict that individuals foraging in rubbish dumps will show differences in clinical and biochemical parameters compared to individuals foraging in a more natural landscape. To test this, we compare clinical and biochemical parameters of black vultures trapped in a rubbish dump, and in a pristine site in the Patagonian steppe.

## Materials and Methods

### Study area

We conducted the study in Northwest Argentine Patagonia (Neuquén and Rio Negro provinces) (around 41°S and 71°W). This area is a typical Patagonian steppe dominated by grasses and shrubs (Cabrera, 1971). In this area, there are farms dedicated mainly to extensive sheep or cattle raising (Mueller & Cueto, 2005). Added to this, there is an important amount of introduced mammals such as red deer (*Cervus elaphus*), wild boar (*Sus scrofa*) and European hare (*Lepus europaeus*) (Navas, 1987). This area has low human densities, large unpopulated areas, but there are important human settlements like San Carlos de Bariloche (with more than 120,000 inhabitants) and Villa La Angostura (with almost 18,000 inhabitants) (INDEC, 2010). These urban settlements produce important quantities of waste that are discarded into two rubbish dumps (Bariloche’s and Villa La Angostura’s rubbish dumps). In the case of Bariloche’s rubbish dump, availability of organic waste has been greatly reduced in the last years, and bird species have stopped using it as a food source. However, in the rubbish dump of Villa La Angostura, organic waste has increased in recent years and there is an important population of birds associated to that place, mainly gulls and birds of prey (Frixione et al., 2012).

### Study species

The black vulture is a medium-sized bird scavenger (weight 1.18–1.94 kg, wingspan 1.67 m) that inhabits from North America to the Chubut province in Argentina (Ferguson-Lees & Christie, 2001). This species has no clear sexual dimorphism. Age categories can be differentiated by the head, which is less wrinkled and more bristly and the beak, which is dark-tipped in immature individuals (Ferguson-Lees & Christie, 2001). Nowadays it is classified as a species of least concern and their population is increasing (IUCN, 2017). Black vultures feed on carcasses of different species like sheep, cattle, wild pig, red deer hares and they also exploit organic matter in sites like rubbish dumps (Iñigo Elias, 1987; Ferguson-Lees & Christie, 2001; Ballejo & De Santis, 2013; Ballejo et al., in press).

It is known that the movement pattern of this species is mainly between their roosting sites and their food source (Novaes & Cintra, 2013), and they tend to be faithful in terms of movement to the food source area (DeVault et al., 2004; Novaes & Cintra, 2015). Moreover, the home range reported for this species is less than 35 km^2^ (Holland et al., 2017). Therefore, individuals roosting close to the cities feed mainly on anthropogenic debris (rubbish dumps and slaughterhouses), and probably spend most of their foraging time in these sites. On the contrary, individuals roosting in the Patagonian steppe spend most of their time in this landscape looking for, and consuming mainly carcasses of ungulates and hares (Ballejo & De Santis, 2013).

### Capture and samples collection

In April and December from 2015 to 2017, we trapped adult black vultures in two different sites of North West Patagonia (1) Dump site: Inside the rubbish dump of Villa La Angostura (Neuquén province, 40°49′S–71°34′W) (2) Steppe site: The Patagonian steppe (Río Negro province, 41°13′S–71°04′W) between 55 and 60 km away from the rubbish dump of Villa La Angostura. To trap the Black Vultures we used walk-in traps and a cannon net baited with meat and bones (Bird et al., 2007). We immobilized the birds after capture in special bags, and before taking the samples we did a clinical examination and took morphological measurements.

We evaluated the following clinical parameters: body mass and dehydration status. After that, a blood sample (3.5 ml) was extracted from the brachial vein; it was then transferred to vials containing dry heparin and transported in a cooler to the lab at 4 °C. On the day of collection, blood samples were centrifuged at 13,000*g* for 10 min to obtain plasma, which was refrigerated at 4 °C until analysis. To determine if foraging in rubbish dumps affects the health status, we compared clinical and biochemical parameters between individuals trapped in the dump site and individuals trapped in the steppe site.

To give more support to the data available that suggests black vultures forage close to their communal roosts and to test the possibility that birds move between sites, we tagged the birds with wing tags. We also used wing tags and microchips to avoid re-sampling of individuals. We then made observations in the rubbish dump, and in the steppe to confirm the presence of tagged birds. We visited the rubbish dump once a week and spent 1 h of observation between April 2016 and 2017. We visited the steppe 16 days and made 10 h of observations per day. We also registered information provided by other observers that reported the location of tagged birds. All the capture and handling methods were approved by the Centro Ecología Aplicada Neuquén (Resolución N° 0263/16, 0049/17 and 1192/17) and Secretaría de Medio Ambiente de la Provincia de Río Negro (Disposición N° 018/16).

### Clinical parameters

A digital balance (portable electronic scale, WeiHeng®, Shijiazhuang, China) was used to record the body mass in each individual trapped. To determine hydration status, we evaluated the refilling time of the brachial vein, the elasticity of the skin and eye characteristics, according to Samour (2008). Hydration status was classified as normal when all the measures look normal and abnormal when these measures look altered (e.g. increase in the time of refilling of the brachial vein, loss of skin elasticity and eyes with sunken aspect).

### Blood parameters

We evaluated the following blood parameters: haematocrit, haemoglobin, uric acid, urea, albumin, total proteins, calcium, glycaemia, globulins, albumen–globulin ratio and hepatic enzymes ALT (alanine aminotransferase), AST (aspartate aminotransferase), AP (alkaline phosphatase) and CPK (creatine phosphokinase). To measure haematocrit we used the standard micro haematocrit method centrifuging a blood micro capillary tube at 1,000*g* during 5 min (Samour, 2008). Haemoglobin was measured in a Roche KX21 analyser. The other parameters were evaluated in a Mindray B200 chemistry analyser. Globulins were estimated through the subtraction of the albumins to the total proteins. The albumin–globulin ratio was computed through the division between albumin fraction and globulin fraction (Cray & Tatum, 1998).

### Statistical analyses

We first performed descriptive statistics to describe values (mean, SD and maximum and minimum values) of the different parameters discriminated by the origin of individuals (dump site and steppe site). To evaluate the differences in hydration status between sites we used Fisher’s exact test. To evaluate the differences in mean value between sites of the different biochemical parameters studied and body mass, we used the Wilcoxon test or *T* test according to the results of the normality test (Shapiro–Wilk test). Finally, to evaluate if there is an effect of site in the concentration values of biochemical parameters we performed a principal components analysis (Dunteman, 1989). All the statistical analysis were performed with R core team version 3.2.3 (R Core Team, 2015) and we consider *p* < 0.05 as significant.

## Results

We sampled 94 adult black vultures, 48 from the rubbish dump of Villa La Angostura (dump site) and 46 from the Patagonian steppe (steppe site). We found that body mass was higher in individuals from the dump site than in individuals from steppe site (body mass dump 2.25 kg ± 0.18, body mass steppe 2.17 kg ± 0.14, *T* = 2.10, *p* = 0.01). Moreover, there were more individuals clinically dehydrated in the steppe site than in the dump site (Fisher’s exact test, *p* = 0.02).

**Figure 1:**
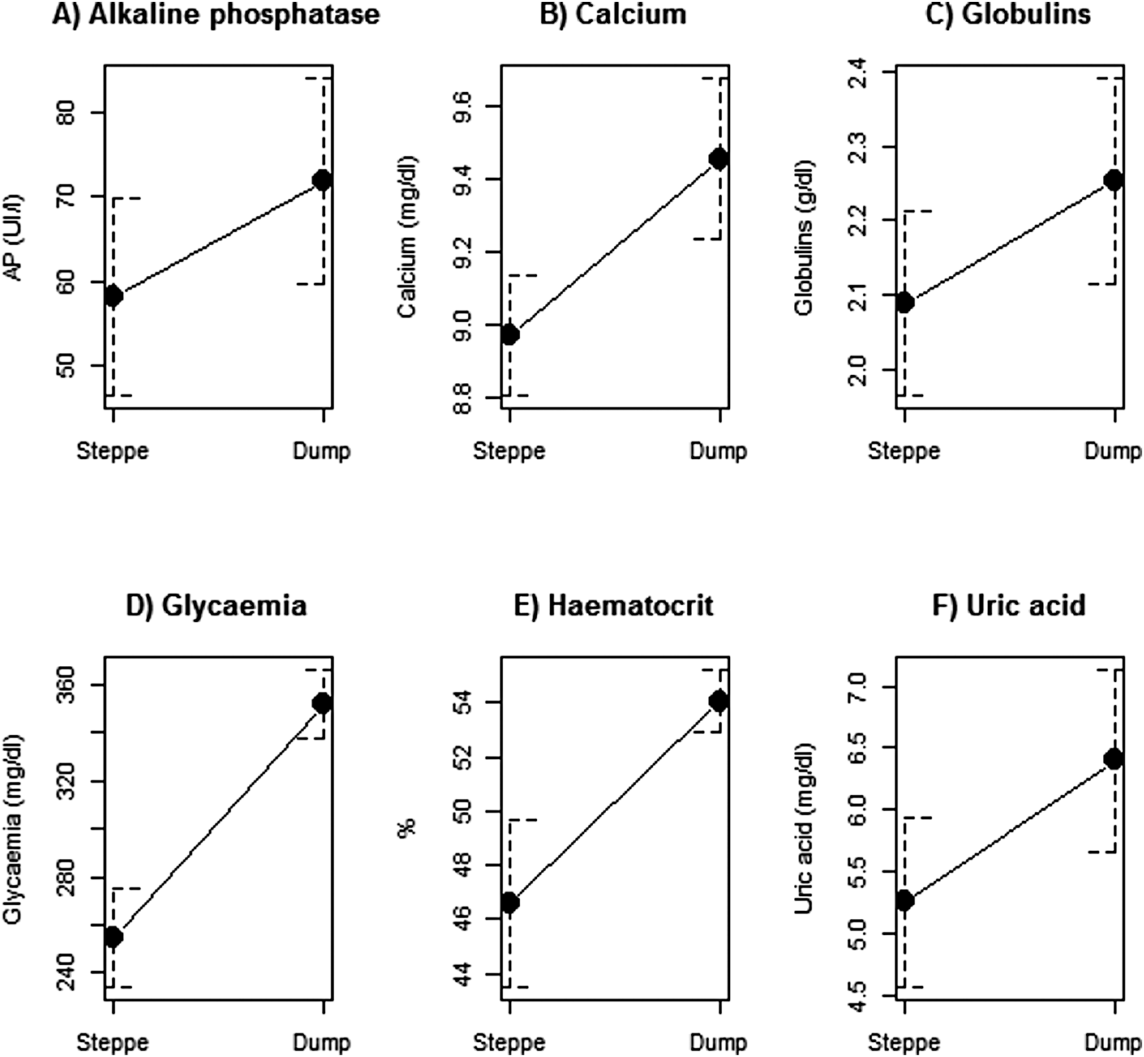
Biochemical parameters that had significantly higher concentration levels in black vultures trapped in a rubbish dump from Patagonia. (A) Alkaline phosphatase, (B) Calcium, (C) Globulins, (D) Glycaemia, (E) Haematocrit, (F) Uric acid.

**Figure 2:**
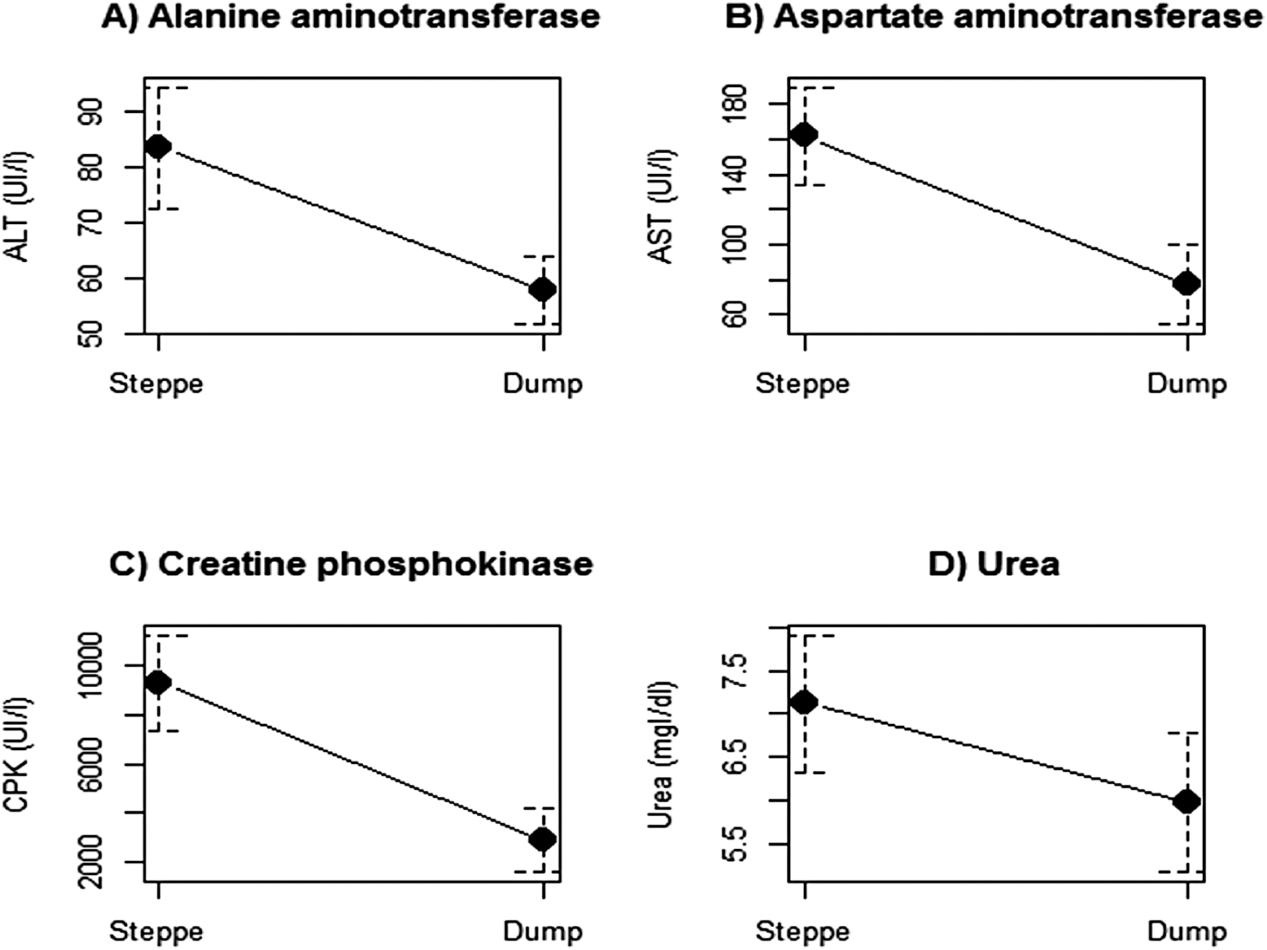
Biochemical parameters that had significantly higher concentration levels in black vultures trapped in the wild area in the Patagoninan steppe. (A) Alanine aminotransferase, (B) Aspartate aminotransferase, (C) Creatine phosphokinase, (D) Urea.

Biochemical parameters differed between the two sites. The mean values of uric acid, calcium, AP, glycaemia, globulins and haematocrit were higher in individuals trapped in the dump site (Table 1; Fig. 1). On the contrary, the hepatic enzymes AST, ALT, CPK and urea were higher in individuals from the steppe site (Table 1; Fig. 2). There were no differences between sites in albumin, haemoglobin, total proteins and albumin–globulin ratio (Table 1).

**Table 1:** Biochemical parameters (mean, SD and range) for black vultures (*Coragyps atratus*) trapped in the rubbish dump (dump site) and in the Patagonian steppe (steppe site).

Parameter	Dump site			Steppe site			Mean difference
	Mean ± SD	*n*	Range	Mean ± SD	*n*	Range	Dump vs. steppe
**Uric acid (mg/dl)**	6.39 ± 2.49	47	2.15–13.9	5.20 ± 2.15	40	2.13–14.4	*W* = 1198.5, *p* = 0.01*
**Albumin (g/dl)**	1.60 ± 0.18	48	1.25–2.10	1.58 ± 0.27	44	0.8–2.52	*W* = 1,072, *p* = 0.90
**Calcium (mg/dl)**	9.45 ± 0.75	48	8–11.6	8.97 ± 0.51	40	8–10.6	*W* = 1369.5, *p* = 0.0002*
**CPK (UI/l)**	2855.5 ± 4424.4	47	466–27,432	9312 ± 6072.3	39	1,200–29,810	*W* = 192, *p* < 0.0001**
**AP (UI/l)**	71.77 ± 41.82	48	18–209	58.13 ± 38.48	44	19–181	*W* = 1,360, *p* = 0.008*
**Globulins (g/dl)**	2.25 ± 0.47	48	1.70–3.90	2.08 ± 0.4	44	0.86–3.15	*W* = 1296.5, *p* = 0.03*
**Glycaemia (mg/dl)**	351.58 ± 49.40	48	236–459	254.47 ± 66.91	44	128–445	*T* = 7.86, *p* = < 0.0001*
**AST (UI/l)**	77.16 ± 79.77	48	27–446	161.88 ± 92.13	44	40–471	*W* = 334, *p* < 0.0001**
**ALT (UI/l)**	57.77 ± 21.1	48	17–121	83.43 ± 35.79	44	24–161	*W* = 567.5, *p* < 0.0001**
**Haemoglobin (g/dl)**	17.58 ± 0.95	47	15.4–19.5	17.77 ± 1.80	44	13.5–22.5	*T* = −0.638, *p* = 0.52
**Haematocrit (%)**	54.04 ± 3.86	47	44–61	46.58 ± 10.1	44	30–62	*W* = 1,417, *p* = 0.001*
**Alb/Glob ratio**	0.72 ± 0.10	48	0.45–0.91	0.80 ± 0.35	44	0.25–2.9	*W* = 899.5, *p* = 0.1
**Total proteins (g/dl)**	3.85 ± 0.59	48	3.15–5.80	3.67 ± 0.45	44	2.70–4.95	*W* = 1223.5, *p* = 0.09
**Urea (mg/dl)**	5.97 ± 2.80	48	3–16	7.12 ± 2.48	40	3–14	W = 617.5, *p* = 0.001**

**Notes:**

* Greater in the dump.

** Greater in the steppe.

**Figure 3:**
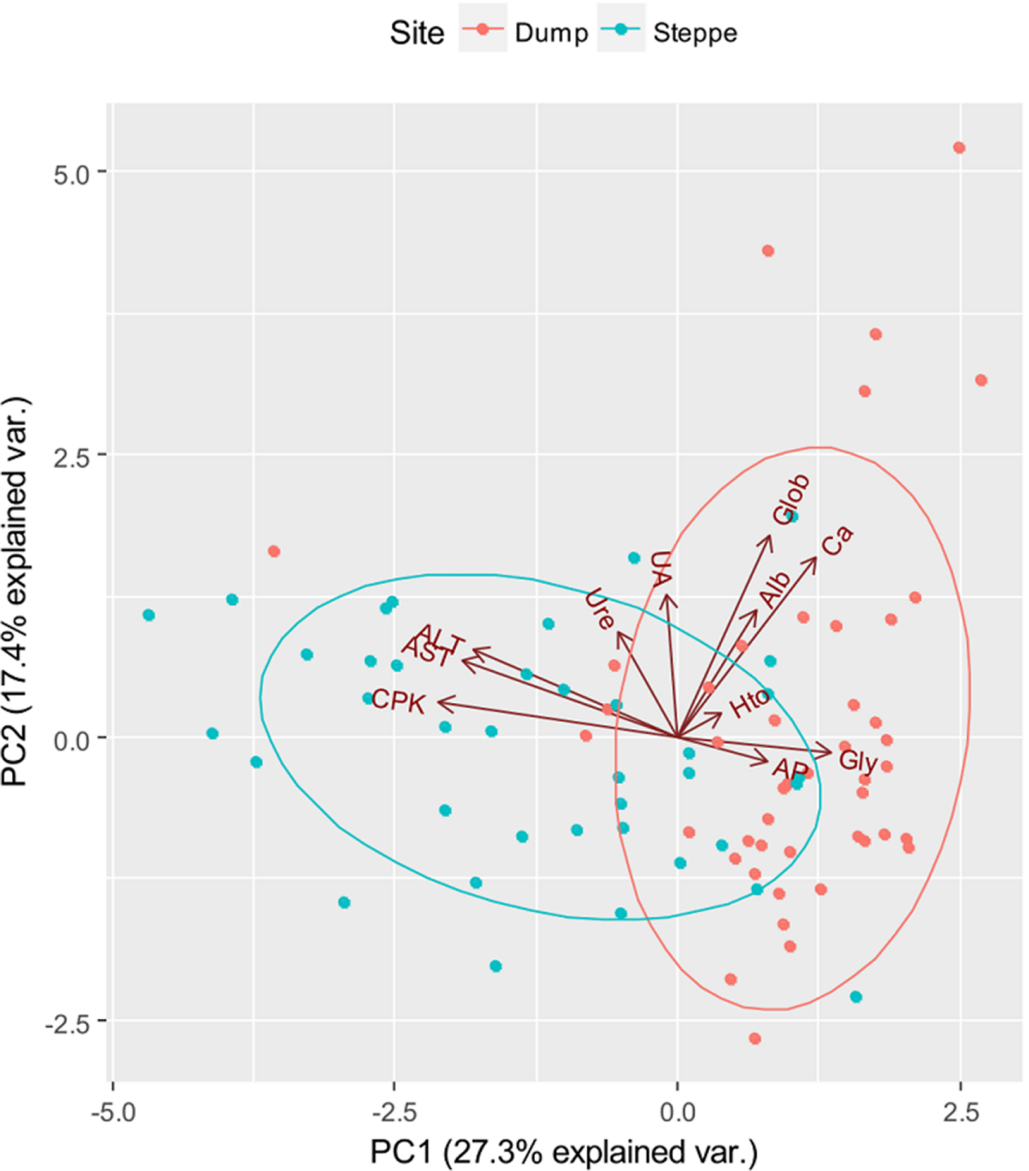
Two first axes of the Principal Component Analysis that ordered individuals by their biochemical parameters. AST (Aspartate aminotransferase), ALT (Alanine aminotransferase), CPK (Creatine phosphokinase), Alb (Albumin), Glob (Globulins), Hto (Haematocrit), Gly (Glycaemia), AP (Alkaline phosphatase), Ca (Calcium), and UA (Uric acid). Red dots correspond to birds trapped in the rubbish dump and light blue dots correspond to birds trapped in the Patagonian steppe.

When we evaluated if biochemical parameters separated the groups by their origin, we found two groups of individuals in the first two axes that respond quite well to the origin but with some overlap (Table 2; Fig. 3). The first group was composed by individuals trapped in the steppe, which are segregated mainly by the following variables CPK, AST, ALT and urea. The second group was composed by individuals trapped in the dump site, which are segregated mainly by the following variables: globulins, glycaemia, AP, haematocrit and calcium.

**Table 2:** Variable loadings for the first four eigenvectors (CP1, CP2, CP3 and CP4) from the principal component analysis performed with biochemical variables of black vultures (*Coragyps atratus*) trapped in two foraging areas.

Parameters	CP1	CP2	CP3	CP4
**Uric acid**	−0.02144807	0.38640272	−0.01971501	0.61605868
**Albumin**	0.16840293	0.34330430	0.28251079	−0.35192106
**Alanine aminotransferase**	−0.43986384	0.23839509	−0.20987946	−0.17528689
**Alkaline phosphatase**	0.19323124	−0.06398857	0.19268915	−0.51048794
**Aspartate aminotransferase**	−0.46156708	0.20969073	−0.14411323	−0.17528689
**Calcium**	0.30046898	0.48587286	−0.05574199	−0.21600459
**CPK**	−0.51341251	0.09240471	−0.07946172	−0.22678599
**Globulins**	0.20065624	0.54534141	−0.12566362	−0.05273312
**Glycaemia**	0.32956143	−0.04285755	−0.36995375	0.09368761
**Hematocrit**	0.09320060	0.06659838	−0.65600818	−0.03531979
**Urea**	−0.12603064	0.28223694	0.47479118	0.27878543
**Proportion of variance**	0.2729434	0.1735840	0.1533919	0.09625357
**Cumulative proportion**	**0.2729434**	**0.4465273**	**0.5999192**	**0.69617278**

Finally, in all the 48 surveys we performed from April 2016 to April 2017 in the dump site we observed at least three black vultures from the 48 that were wing tagged in this site. From the 29 birds wing tagged in the Patagonian steppe just in three cases (three different birds) visited the rubbish dump of Villa La Angostura during the same observation period. On the other hand, in 16 surveys we did in the steppe just one individual tagged in the rubbish dump was observed in the Patagonia steppe. Then, there are some movement between areas but it seems that birds eating in the rubbish dump remain in those areas.

## Discussion

Our results show clear differences in some clinical and biochemical parameters studied between individuals trapped in the rubbish dump and individuals trapped in the wild steppe. There are differences in body mass and clinical hydration status, which can be considered indicators of general condition. We also found clear differences in some biochemical parameters between black vultures from the dump and the steppe sites, which could suggest incipient and potentials health alterations. While there is some movement between the feeding habitats, individuals tagged in the rubbish dump appear to stay most of the time there foraging on organic waste. Therefore, our results suggest that the site selected for foraging can influence the values of clinical and biochemical parameters and, thus, the health of individuals.

Black vultures trapped in the dump have greater body mass than individuals from the steppe. This suggests that individuals from the dump, which access easily to great quantities and high predictable food source spending little energy, increase their body mass more than birds that feed from wild areas. The same results have been observed in gulls using organic waste as food source (Auman, Meathrel & Richardson, 2008). In fact, it is known that predictability in food sources may influence body mass in birds, being unpredictability associated to body mass loss (Cucco et al., 2002). The higher body mass found in individuals from the dump could be interpreted as a positive effect of this kind of food subsidy (Plaza & Lambertucci, 2017). This is because body mass, an indicator of body condition (Seewagen, 2008; Schamber, Esler & Flint, 2009), is related with survival rate, reproductive fitness and health status (Hepp et al., 1986; Bergan & Smith, 1993; Peig & Green, 2009). In addition, individuals from the dump showed fewer alterations in the clinic hydration status. This could be because individuals from the steppe, unlike the individuals from the dump site, spend more time flying searching for food, which can produce more metabolic effort (Norberg, 1977) producing flight incurred dehydration (Carmi et al., 1993; Giladi & Pinshow, 1999).

Individuals trapped in the dump site showed greater values of uric acid, calcium, AP, glycaemia, globulins and haematocrit than individuals trapped in the steppe. The difference between sites in these parameters could be related to the characteristics of the food present in the rubbish dump (Parfitt, Barthel & Macnaughton, 2010), and to the high presence of pathogens in these sites (Flores-Tena et al., 2007; Matejczyk et al., 2011). For instance, acid uric is related to protein metabolism, and an increase in its value is directly related to a protein ingestion increase (Lumeij & Remple, 1991; Samour, 2008; Krautwald-Junghanns, Orosz & Tully, 2008). This can be the case of individuals present in the rubbish dump that could ingest great quantities of proteins on a daily basis as a consequence of the high availability of this nutrient in this site (Parfitt, Barthel & Macnaughton, 2010). However, the increment in uric acid could also indicate potential kidney damage probably as a consequence of the high ingestion of proteins (Chandra et al., 1984). The higher values of AP in individuals from the dump can be difficult to interpret because this enzyme is present in different organs like bone, liver and intestine and can increase its values due to different processes taking place in these organs, both, physiologically and pathologically (Franson, Murray & Bunck, 1985; Joseph, 1999; Krautwald-Junghanns, Orosz & Tully, 2008). Calcium, glycaemia and haematocrit can also be influenced by the peculiarity of this diet, especially due to its predictability and the high availability (Cucco et al., 2002; Fair, Whitaker & Pearson, 2007). For instance, individuals foraging in the rubbish dump may eat more carbohydrates in form of human preparations (snacks, cookies and cereals, among others), which are discarded there (Parfitt, Barthel & Macnaughton, 2010). High ingestion of carbohydrates may produce increased glycaemia levels since metabolism in birds of prey is not well prepared to ingest an excess of this nutrient due to the continuous process of gluconeogenesis that they exhibit (Migliorini et al., 1973; Myers & Klasing, 1999; Pollock, 2002). The same situation can occur with calcium levels, which may be associated to the great availability of bones or calcium/vitamin D-fortified dairy products like cheese that exist in rubbish dumps (Parfitt, Barthel & Macnaughton, 2010). Finally, the greater levels of globulins (immune proteins) could be related to the high presence of pathogens in dumps, which can trigger an acute phase proteins response (Gruys et al., 2005). Therefore, foraging in rubbish dumps may help to increase the weight but could be producing nutritional problems, more risk of infections and could be affecting in the long term, the normal function of the kidney.

Individuals trapped in the wild steppe showed higher levels of hepatic enzymes (AST, ALT and CPK) and urea. AST and ALT can indicate liver damage (Franson, Murray & Bunck, 1985; Samour, 2008; Krautwald-Junghanns, Orosz & Tully, 2008). The increments of these hepatic enzymes in individuals from the Patagonian steppe may be explained by the health impact that different toxics like lead or pesticides produce on liver functioning. In this sense, lead in the form of ammunitions produced by hunting activities and pesticides that may be produced by fish farms are common in this area (Lambertucci et al., 2011; Martínez-López et al., 2015; Wiemeyer et al., 2017). The higher levels of CPK in individuals from the steppe, which increase due to muscle damage, are difficult to explain because this enzyme increments its values due to a very slight damage (Nyska et al., 1994; Krautwald-Junghanns, Orosz & Tully, 2008) and thus can be associated with multiple causes like for instance the trauma associated with fights due to the high competition for carcasses that is common in the steppe (Carrete et al., 2010), but not in the rubbish dump, where food supply is constant. Added to this, the higher values of CPK can be produced by the handling method (Bollinger et al., 1989). Finally, the higher values of urea can be related to flight incurred dehydration (as explained above), which could increment the values of this parameter (Giladi & Pinshow, 1999; Krautwald-Junghanns, Orosz & Tully, 2008).

Considering all the variables together, we found that the birds from the dump and the steppe differed in their biochemical parameters forming two groups, but with some overlapping and variability. One group was composed by individuals trapped in the rubbish dump, which were explained mainly by higher values of glycaemia, calcium, globulins, uric acid, haematocrit and AP. The other group was composed by the individuals trapped in the steppe, which had high values of CPK, ALT, AST and urea. Some overlapping of individuals between these groups could be explained by the fact that some birds move between those places, as it was observed. This can happen, for instance, when there is a lack of carcass availability in the steppe and individuals use organic waste as a buffer food, as has been observed in other species (Olea & Baglione, 2007).

## Conclusion

Our results show that foraging in rubbish dumps can influence some clinical and biochemical parameters of scavenger birds. We show that the use of organic waste appears to be positive when considering some typical variables used to study the health of wildlife animals (e.g. body mass and haematocrit). However, the apparently good health condition observed with some variables could be masking actual problems that can threat individuals in the future. Moreover, this study does not consider pathogens and toxics present in the dump site, which can also produce severe health alterations that need to be considered in future research. There are several species exploiting organic waste around the world, including threatened species (Plaza & Lambertucci, 2017). For instance, endangered species as the California condor (*Gymnogyps californianus*) in the USA (Rideout et al., 2012), the Andean condor (*Vultur gryphus*) in Chile (Pavez, 2014) and the Egyptian vultures (*Neophron percnopterus*) in Africa (Gangoso et al., 2012) forage in rubbish dumps. Therefore, our results on an abundant vulture can be regarded a call for considering the potential negative effects that these food subsidies have on other species, particularly on threatened species.

## Supplemental Information

10.7717/peerj.4645/supp-1 Supplemental Information 1 Raw data.

## Additional Information and Declarations

### Field Study Permissions

The following information was supplied relating to field study approvals (i.e. approving body and any reference numbers): Field experiments were approved by the Centro ecología aplicada Neuquén (CEAN) and Secretaria de ambiente de Río Negro.

### Data Availability

The following information was supplied regarding data availability: The raw data are provided in Supplemental Dataset Files.
